# Novel Latex Microsphere Immunochromatographic Assay for Rapid Detection of Cadmium Ion in Asparagus

**DOI:** 10.3390/foods11010078

**Published:** 2021-12-29

**Authors:** Naifeng Xu, Qiaojuan Zhu, Jiangxiong Zhu, Jingze Jia, Xinlin Wei, Yuanfeng Wang

**Affiliations:** 1 Institute of Engineering Food, College of Life Sciences, Shanghai Normal University, 100 Guilin Road, Xuhui District, Shanghai 200234, China; xunaifeng217@163.com (N.X.); zhuqiaojuan2333@163.com (Q.Z.); zjx261023@163.com (J.Z.); jhappy100@163.com (J.J.); 2 Department of Food Science & Technology, School of Agriculture and Biology, Shanghai Jiao Tong University, 800 Dongchuan Road, Minhang District, Shanghai 200240, China

**Keywords:** cadmium ion, monoclonal antibody, immunochromatography, latex microspheres, asparagus

## Abstract

Recently, concerns about heavy metal cadmium ion (Cd^2+^) residue in asparagus have been frequently reported, and there is an urgent need to develop an effective, sensitive, and rapid detection method for Cd^2+^. In this study, we innovatively combined molecular microbiology to carry out the comparative screening of Cd^2+^ chelators in a green, efficient, and specific way. The knock-out putative copper-transporter gene (*pca1*Δ) yeast strain with high sensitivity to Cd^2+^ was first used to screen the Cd^2+^ chelator, and the optimum chelator 1-(4-Isothiocyanatobenzyl)ethylenediamine-N,N,N,N′-tetraacetic acid (ITCBE) was obtained. Additionally, a rapid latex microsphere immunochromatographic assay (LMIA) was developed, based on the obtained monoclonal antibody (mAb) with high specificity and high affinity (affinity constant Ka = 1.83 × 10^10^ L/mol), to detect Cd^2+^ in asparagus. The 50% inhibitive concentration (IC_50_) of test strip was measured to be 0.2 ng/mL, and the limit of detection (IC_10_) for qualitative (LOD, for visual observation) and quantitative detection (LOQ, for data simulation) of the test strip was 2 ng/mL and 0.054 ng/mL, respectively. In all, the developed mAb-based LMIA shows a great potential for monitoring Cd^2+^ in asparagus, even in vegetable samples.

## 1. Introduction

The pollution of heavy metal ions has brought a heavy burden to the environment due to industrialization [1]. The heavy metal ions releasing into the environment (such as air, soil, and water) have caused global heavy metal pollution and have attracted worldwide attention due to human activities [2]. Among the heavy metals, cadmium has attracted much attention due to its strong toxicity and difficult degradation by microorganisms or organic organisms [3]. The main form of cadmium in the environment is Cd^2+^, and it will migrate into the human body mainly through the enrichment of the food chain [4]. Due to the long half-life of Cd^2+^ accumulated in the body, it can cause the disorder of cell cycle, proliferation, differentiation, DNA replication and repair at the cell level, affect the pathway of apoptosis and interfere with the normal metabolism, and ultimately pose a potential risk to human health [5,6,7,8]. Furthermore, exposure to excessive Cd^2+^ concentrations can reduce the growth rate of crops by affecting water conditions and transpiration, photosynthesis, enzyme activity, absorption, and the translocation of many macro and micro nutrients [9]. According to the investigation of the toxicity of heavy metal Cd^2+^, the International Agency on Cancer Research has defined cadmium as a carcinogen [10]. In 2006, the Codex Alimentarius Commission set the international standard value of cadmium contained in polished rice at 0.4 mg/kg [11]. The U.S. Food and Drug Administration (USFDA) has a regulatory limit of 0.1 mg/kg for cadmium levels in infant formulae [12]. The Codex Alimentarius Commission (FAO/WHO) Expert Committee on Food Additives (JECFA) stipulates that the weekly cadmium intake of adults should be below 5.8 μg/kg [13]. The Chinese national standard stipulates that the cadmium content in fresh vegetables must be less than 0.05 mg/kg (GB 2762-2017). Therefore, it is critical to monitor the Cd^2+^ content in food.

The traditional and classic detection methods of heavy metal cadmium, such as atomic absorption spectroscopy (AAS), atomic fluorescence spectroscopy (AFS), inductively coupled plasma atomic emission spectrometry (ICP-AES), high-performance liquid chromatography (HPLC), X-ray fluorescence spectrometry (XRF), and laser-induced breakdown spectroscopy (LIBS) [14,15,16,17,18,19], have been well applied in the analysis of cadmium. These detection methods possess many advantages such as good accuracy, high sensitivity, low detection limit, and wide detection range. Additionally, they have been widely used in various industries. However, these methods are not suitable for real-time and on-site monitoring and limit their wide use because of the need for expensive instruments and professional operating techniques, as well as complex sample pre-processing and long analytical time. Therefore, there is an urgent need to develop a rapid, economical, reliable, and uncomplicated method to accurately quantify the cadmium level in food.

The immunochromatographic assay (ICA) detection technology has received great attention in the food detection of cadmium, due to advantages including rapid visual convenience, on-site, and low-cost [20]. Compared with the traditional instrument detection, the ICA is considered to be a simple method, can be easily operated without any equipment, and can be applied to the on-site analysis of large-scale samples [21,22]. A large volume of published ICA assays have been used to develop and detect Cd^2+^ levels in various foods. Xing et al. developed an immunochromatographic assay based on the silver-enhanced colloidal gold method, and it could be accurately applied to the Cd^2+^ detection of drinking water [23]. Xiao et al. established an immunochromatographic technique based on quantum dots and gold nano-stars for Cd^2+^ detection, which can be accurately used for the detection of Cd^2+^ in tap water [24]. Fu et al. published an immunochromatographic technique based on surface-enhanced Raman scattering (SERS) to detect Cd^2+^ in tap water, river water, and soil leaching water, which showed high specificity and high recovery [25]. To date, there are few reports on the ICA detection method against Cd^2+^ in vegetables, especially asparagus. In addition, higher identifiable signal intensity is needed to further improve the sensitivity of the ICA method. Most ICA methods using microspheres as detection probes have been reported to show higher sensitivity and lower limit of detection (LOD) than traditional ICA methods [26,27]. Compared with fluorescence immunochromatography or SERS immunochromatography, the microspheres carrying different functional groups in immunochromatography assay have the same particle size, strong stability, and different colors, which can display more sensitive detection results and wider application prospects [28]. At the same time, it can be applied to large-scale applications more conveniently and flexibly without equipment assistance. Recently, there have been more reports indicating excessive heavy metal residues in asparagus [29]. These works prompted us to explore a novel ICA microsphere detection assay to detect Cd^2+^ in asparagus.

In the current study, we explored the binding capability of chelators with specific functional groups to Cd^2+^ through the mutant yeast strain. In addition, the LMIA test strip was developed based on the antigen–antibody specific reaction, and the application of the test strip in Chongming asparagus was preliminarily explored. These results provide a great potential for cadmium monitoring in vegetables.

## 2. Materials and Methods

### 2.1. Materials and Reagents

The wild-type (WT) haploid control yeast *Saccharomyces cerevisiae* strain (BY4741) and *pca1* knock-out mutant (*pca1*Δ) [30] were purchased from the Open Biosystems (Boston, MA, USA). The *pca1* gene has been reported to control the cadmium level in yeast cells, and its mutant strain has excellent cadmium resistance under cadmium stress [31,32]. Asparagus was harvested from Chongming Island (Shanghai, China). Six-week-old female Bagg Albino (BALB/c) mice were purchased from SLAC Laboratory Animal Co., Ltd. (Shanghai, China). Mouse myeloma cells (SP2/0) were obtained from the National Collection of Authenticated Cell Cultures of China (Shanghai, China). The other chemicals used were summarized in Table S1. All other chemicals were of analytical grade unless otherwise specified.

### 2.2. Screening of Chelators for Cd^2+^

The resuscitated WT and *pca1*Δ yeast strains were cultured in Yeast Peptone Dextrose Agar (YPD) solid medium until the colonies grew completely (30 °C, 25–30 h). Next, the colonies were seeded in the YPD liquid medium (3 mL) and incubated overnight (30 °C, 190 rpm) to propagate, and then the optical density under 600 nm (OD_600_) of the expanded medium was measured. To reduce errors, three gradient concentrations were set for each yeast solution, including OD_600_ = 0.1, OD_600_ = 0.02, and OD_600_ = 0.004. The prepared yeast solution (3 µL) was seeded into a 96-well plate and CdCl_2_ (0, 1, 2, 5, 10, and 15 μM) were added. The strain was cultured with YPD solid medium at 30 °C for 3–4 days, and then the growth was recorded. Repeating this step, 8 common chelators with different concentrations were added with the concentration ratio of 2:1, including 2-(5-Bromo-2-Pyridylazo)-5-(Diethylamino)Phenol (5-Br-PADAP) (0, 10, and 20 μM), 2-Mercaptobenzothiazole (2-MBT) (0, 10, 20, and 40 μM), N-Acetyl-L-cysteine (NAC) (0, 10, 20, and 40 μM), 1-(4-nitrophenyl)-3-[4-(phenylazo)phenyl]-1-triazen (CADION) (0, 10, 20, 40, and 50 μM), dimercaptosuccinic acid (0, 10, 20, 40, and 50 μM), CaNa_2_-Ethylene Diamine Tetraacetic Acid (EDTA) (0, 10, 20, 40, and 50 μM), EDTA (0, 10, 20, 40, and 50 μM), and ITCBE (0, 10, 20, 40, and 50 μM), and their structural formulas were shown in Figure S1.

### 2.3. Synthesis of Complete Antigen for Cd^2+^

Based on the screening results, the chelator with the strongest binding capability with Cd^2+^ was used as a hapten for the synthesis of the complete antigen. The difference between the design and synthesis effect of the hapten was explored through the following two methods: (1) The chelator first coupled with Cd^2+^ and then combined with the carrier protein; (2) The chelator first bound to the carrier protein and then bound to Cd^2+^.

#### 2.3.1. Synthesis of Cd^2+^ Immunogen

Two carrier proteins including bovine serum albumin (BSA) and keyhole limpet hemocyanin (KLH) were selected to participate in the synthesis of Cd^2+^ immunogen to obtain the optimum antibody.

##### Carrier Protein BSA and Method 1

The ITCBE solution (300 μL, 10 mg/mL) was mixed with Cd(NO_3_)_2_ (1416 μL, 1 mg/mL) and the pH was adjusted to 7.0 with 1 M NaOH. After that, Cd-ITCBE was obtained by magnetic stirring at room temperature for 6 h. Then, 15.84 mg, 7.40 mg, and 5.00 mg of BSA were dissolved in 1 mL 0.01 M hydroxyethyl piperazineethanesulfonic acid (HBS) buffer (pH = 9.0), respectively. The Cd-ITCBE solution was mixed with the BSA according to the molar ratio of Cd-ITCBE: BSA = 10:1, 20:1, and 30:1, and the pH was adjusted to 9.0. Subsequently, the mixture was magnetically stirred at room temperature for 24 h. Next, it was dialyzed with 0.01 M HBS buffer (pH = 7.4) for 3 days. After dialysis, the immunogen was obtained and stored at −20 °C.

##### BSA and Method 2

A total of 7.92 mg and 3.75 mg of BSA were dissolved in 1 mL 0.01 M HBS buffer (pH = 9.0), respectively. The ITCBE solution was mixed with the above BSA solution (pH = 9.0) according to the molar ratio of ITCBE: BSA = 20:1 and 40:1. The prepared ITCBE-BSA solution was mixed with 472 μL 10 mg/mL Cd(NO_3_)_2_ under pH = 7.4 and then magnetically stirred at room temperature for 6 h. After dialysis, the immunogen was obtained and stored at −20 °C.

##### KLH and Method 1

The ITCBE solution (263 μL, 10 mg/mL) was mixed with Cd(NO_3_)_2_ (1180 μL, 1 mg/mL) and the pH was adjusted to 7.0. The prepared Cd-ITCBE solution was mixed with KLH solution (1364 μL, 6.6 mg/mL) with the molar ratio of Cd-ITCBE: KLH = 3000:1, and the pH was adjusted to 9.0. Next, the mixture was magnetically stirred at room temperature for 24 h. After dialysis, the immunogen was obtained and stored at −20 °C.

##### KLH and Method 2

The ITCBE solution (150 μL, 10 mg/mL) was mixed with 776 μL 6.6 mg/mL KLH (pH = 9.0) with the molar ratio of ITCBE: KLH = 3000:1 and then stirred magnetically at room temperature for 24 h to obtain ITCBE-KLH. The prepared ITCBE-KLH solution was mixed with 672 μL of 1 mg/mL Cd(NO_3_)_2_ (pH = 7.4). Next, the mixture was magnetically stirred at room temperature for 6 h. After dialysis, the immunogen was obtained and stored at −20 °C.

#### 2.3.2. Synthesis of Coating Antigen

The ITCBE solution (300 μL, 10 mg/mL) was mixed with 1416 μL 1 mg/mL Cd(NO_3_)_2_ (pH = 7.0) and then was magnetically stirred at room temperature for 24 h to obtain Cd-ITCBE. A total of 15.84 mg, 7.40 mg, and 5.00 mg of Ovalbumin (OVA) was dissolved in 1 mL 0.01 M HBS buffer (pH = 9.0), respectively. Subsequently, the Cd-ITCBE solution was added into different concentrations of OVA with a molar ratio of 10:1, 20:1, and 30:1, respectively, and the pH was adjusted to 9.0. Next, the mixture was magnetically stirred at room temperature for 24 h. After dialysis, the coating antigen was obtained and stored at −20 °C.

### 2.4. Preparation and Performance Evaluation of mAb

The obtained complete antigen and carrier protein were characterized by sodium dodecyl sulfate polyacrylamide gel electrophoresis (SDS-PAGE) and Ultraviolet (UV) spectra at 200–400 nm, the identification of chelator was carried out by UV spectra at 200–400 nm.

### 2.5. Identification of the Complete Antigen for Cd^2+^

The synthesized immunogen was emulsified with Freund’s complete or incomplete adjuvant, and the emulsified immunogen was injected subcutaneously into the nape and back of mice to produce antibodies against Cd^2+^. The animal experiments were performed following the animal guidelines of the Use Committee of Shanghai Jiao Tong University (approval A2020080). Briefly, 100 μg immunogen was injected into each BALB/c mice for the primary immunization, and 50 μg immunogen was used for the next five booster immunizations. After each booster immunization, mouse serum was collected and the titer and inhibition rate for antibodies were determined by indirect enzyme linked immunosorbent assay (ic-ELISA). Next, mice producing more efficient antibodies were selected and injected intraperitoneally for 25 μg immunogen. Then, the mouse spleen was collected, the mouse spleen cells were separated, and the obtained spleen cells were fused with SP 2/0 cells to produce hybridoma cells.

The target hybridoma cells were screened with HAT (Hypoxanthine, Aminopterin, and Thymidine) and HT (Hypoxanthine and Thymidine) selection mediums by the ic-ELISA method. Briefly, the cells after 3 days of fusion were seeded into a 96-well plate and a 100 μL HAT selection medium was added. After culturing for 48 h, the original culture medium was replaced with 270 μL of HT selection medium and was continued to incubate for 48 h. Next, according to the titer and inhibition rate of the immune serum, the ic-ELISA method was employed to detect the positive cell fusion, and the HBS buffer containing 1 mM EDTA was used as the standard to evaluate the inhibitory effect of EDTA-Cd. Subsequently, the positive cells were subcloned 4 times by the limiting dilution method to obtain a monoclonal cell line. The ascites were induced by intraperitoneal injection of hybridoma cells into BALB/c mice to achieve the mass production of monoclonal antibodys (mAbs). Purification of mAbs in ascites was performed by the caprylic acid-ammonium sulfate precipitation method [33]. Briefly, A total of 0.5 mL of ascites was mixed with 1 mL of acetic acid-sodium acetate buffer (molar ratio = 6:22), and then 16.5 μL of n-octanoic acid (99% purity) was added. Subsequently, the mixture was stirred at room temperature for 30 min and stood at 4 °C for 2 h. After centrifugation for 30 min (8000 rpm, 4 °C), the supernatant was dialyzed (cutoff Mw 20 kDa) with 0.01 M phosphate buffered saline (PBS) (pH = 8.0) for 24 h. Next, 1.5 mL of saturated ammonium sulfate (pH = 7.4) was added and then stood at 4 °C for 12 h. After centrifugation for 30 min (8000 rpm, 4 °C), the precipitate was re-dissolved in 0.01 M PBS buffer (pH = 7.4) and then dialyzed (cutoff Mw 20 kDa) with 0.01 M PBS buffer (pH = 8.0) for 72 h. The retentate was centrifugated for 30 min (4 °C, 8000 rpm) and the supernatant was obtained and stored at −20 °C.

The concentration of mAb was determined by the BCA protein assay kit (Solarbio, Beijing, China), the subtype of the mAb was identified by the Mouse monoclonal antibody subtype identification (MAI) ELISA kit (Proteintech, Wuhan, China), and the purity of mAb was carried out by the SDS-PAGE method. The antibody titer and affinity were determined by the non-competitive ic-ELISA regarding the method reported by Liu et al. [27].

### 2.6. Cross-Reactivity of mAb for Cd^2+^

The IC_50_ of the obtained mAb and EDTA-metal ions (including Pb^2+^, Hg^2+^, As^3+^, Cu^2+^, Fe^3+^, Ni^+^, Cr^3+^) was measured by the ic-ELISA, and the cross-reactivities of the obtained mAb were calculated according to the following formula.

Cross-reactivity (CR, %) = IC_50_ of EDTA-metal ions/IC_50_ of EDTA-Cd^2+^ (1)

### 2.7. Preparation of Latex Microsphere Immunochromatographic Test Strip for Cd^2+^

The detection probe was synthesized by coupling the mAb against EDTA-Cd^2+^ with the highest titer and the optimum inhibition rate with red carboxyl microspheres (300 nm). Briefly, 1 mL microspheres and an appropriate amount of 1-(3-Dimethylaminopropyl)-3-ethylcarbodiimide hydrochloride (EDC)/N-Hydroxysuccinimide (NHS) mixture (molar ratio = 1:1) were added to 9 mL of 2-(N-Morpholino)ethanesulfonic acid monohydrate (MES) buffer (pH = 5.0) and were activated in a shaker at 37 °C for 30 min. After centrifugation at 14,000 rpm, the supernatant was removed and 10 mL PBS was added. After mixing, 200 μg mAb was added and incubated for 2 h in a shaker at 37 °C. Next, 1 mL 5% BSA solution was added and the mixture was blocked in a shaker at 37 °C for 30 min. After centrifugation, the supernatant was removed and 5 mL microsphere storage solution was added and stored at 2–8 °C. The coating antigen (0.09 mg/mL, as capture reagent) was sprayed on the test (T) line and 1 mg/mL Goat anti-mouse IgG antibody was sprayed on the control (C) line. The test strip was cut into 4 mm uniform strips and stored in a drying condition for further use.

### 2.8. Sensitivity Evaluation of Test Strips

The HBS-EDTA solution (pH = 9.0) was used to dilute 1 mg/mL Cd^2+^ standard solution into a series of concentration gradients, including 0, 0.2, 0.5, 1, 2, 5, 10, and 20 ng/mL, then they were determined by the prepared LMIA test strip. The ImageJ (Version 1.8.0) software was used to read the gray value of T and C lines, and a standard curve was established.

### 2.9. Specificity Evaluation of Test Strips

The sensitivity of the other seven common heavy metal ions on the test strip was determined based on the cross-reactivity of mAbs with the optimum immune effect. Hg^2+^ standard solution was diluted to 5 ng/mL and 50 ng/mL with HBS-EDTA solution (pH = 9.0), the other six heavy metal ions were diluted to 50 ng/mL and 500 ng/mL, and they were determined by the prepared LMIA test strip.

### 2.10. Sample Treatment and Determination

Asparagus samples (verified as negative samples by atomic absorption spectrophotometry) were pre-treated according to the pre-treatment method by Wang et al. with slight modification [34]. Briefly, the dried sample was crushed, and then Cd^2+^ standard solution (50 μL, 1 mg/mL) was added to sample powder (0.5 g), and the negative control was regarded without Cd^2+^ standard solution. A total of 10 mL of 65% HNO_3_ was added to the sample and stood at room temperature overnight. After boiling, the pH was adjusted to 7. Subsequently, the sample solution was diluted with HBS-EDTA buffer (pH = 9.0) in multiples of 2 times (×2), 10 times (×10), 20 times (×20), and 50 times (×50). The prepared test strips were used to determine the Cd^2+^ content of each dilution group and the negative control group. Repeating the above steps, the optimum dilution multiple was selected for further detection of the samples containing different concentrations of Cd^2+^ (0, 2, 10, 50, and 100 ng/mL).

### 2.11. Statistical Analysis

All experimental statistical data were expressed as the mean ± standard deviation. All data analysis was performed by using Origin 7.0 software (Microcal, Northampton, MA, USA).

## 3. Results and Discussion

### 3.1. Screening of Chelators for Cd^2+^

**Figure 1:**
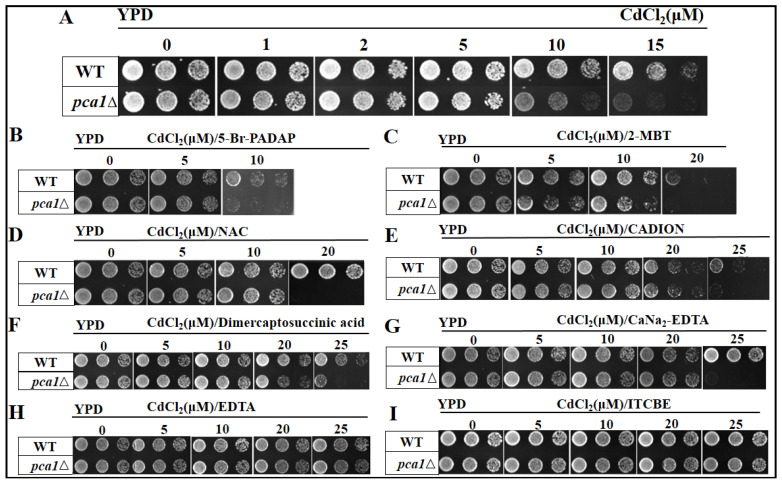
The growth of WT and *pca1*Δ yeast strains incubated with various chelators plus distinct concentrations of Cd^2+^. (**A**) Different concentrations of Cd^2+^ (0, 1, 2, 5, 10, and 15 μM); (**B**) Different concentrations of Cd^2+^ (0, 5, and 10 μM) plus 5-Br-PADAP (0, 10, and 20 μM); (**C**) Different concentrations of Cd^2+^ (0, 5, 10, and 20 μM) plus 2-MBT (0, 10, 20, and 40 μM); (**D**) Different concentrations of Cd^2+^ (0, 5, 10, and 20 μM) plus NAC (0, 10, 20, and 40 μM); (**E**) Different concentrations of Cd^2+^ (0, 5, 10, 20, and 25 μM) plus CADION (0, 10, 20, 40, and 50 μM); (**F**) Different concentrations of Cd^2+^ (0, 5, 10, 20, and 25 μM) plus dimercaptosuccinic acid (0, 10, 20, 40, and 50 μM); (**G**) Different concentrations of Cd^2+^ (0, 5, 10, 20, and 25 μM) plus CaNa_2_-EDTA (0, 10, 20, 40, and 50 μM); (**H**) Different concentrations of Cd^2+^ plus EDTA (0, 10, 20, 40, and 50 μM); (I) Different concentrations of Cd^2+^ (0, 5, 10, 20, and 25 μM) plus ITCBE (0, 10, 20, 40, and 50 μM).

Cd^2+^ does not have T and B cell epitopes and cannot directly induce the body to produce specific antibodies due to its simple structure. According to the well-known theory of hapten immunity, Cd^2+^ must be chelated to synthesize the artificial antigen. A large number of studies show that macromolecular bifunctional chelators are the most ideal couplers such as EDTA and its derivatives [35]. Figure 1A provides the growth of WT and *pcal*Δ yeast strains (three culture concentrations) in different concentrations of Cd^2+^. As can be seen, the growth of the *pca1*Δ yeast strain was significantly inhibited by 10 μM Cd^2+^ and was completely inhibited by 15 μM Cd^2+^, while the WT strain was not affected by Cd^2+^ toxicity. The previous report indicated that the yeast mutant strain carrying *pca1*Δ was intolerant to Cd^2+^ and showed the specificity (only sensitive to Cd^2+^) [32], our results further confirmed this report. If the added chelators can chelate a certain amount of Cd^2+^, the growth of the *pca1*Δ yeast strain can be partially or completely restored. Based on this, the binding capability of different chelators to Cd^2+^ was compared. The chelator with the optimum chelating effect was selected through the recovery growth of the *pca1*Δ yeast strain for the synthesis of complete antigen for Cd^2+^. As shown in Figure 1B–I, eight common chelators could chelate a certain amount of Cd^2+^ and increased Cd^2+^ tolerance in *pca1*Δ yeast strains, but their chelating capacity was significantly different. Therein, three chelators including 5-Br-PADAP, 2-MBT, and NAC had the limited chelating effect and could not cope with 20 μM Cd^2+^. CADION, dimercaptosuccinic acid, and CaNa_2_-EDTA had good chelating effects and the *pca1*Δ yeast strains recovered their growth in the media contaminated by 20 μM Cd^2+^. EDTA and ITCBE exhibited the optimum chelating capability, the *pca1*Δ yeast strains could grow well at 0–25 μM Cd^2+,^ and its growth was not affected by Cd^2+^ additions. Given the special structure of EDTA and its limited capability to couple carrier proteins (only exposed carboxyl groups), ITCBE was selected for the synthesis of complete antigen for Cd^2+^. The current experiment of gene-knockout yeast strain intuitively explained why ITCBE had been used as a bifunctional chelator in the immunodetection of Cd^2+^ [20,36,37]. In addition to our current research, other reports also indicate that yeast strains with special genotypes could be used to investigate Cd^2+^ tolerance [38,39]. Additionally, we innovatively used yeast mutants to screen chelators against Cd^2+^, which has never been reported in previous studies. Consistently, this method also showed satisfactory results and might be applied to the screening of chelators with better characteristics in the future.

### 3.2. Synthesis and Identification of Complete Antigens for Cd^2+^

**Figure 2:**
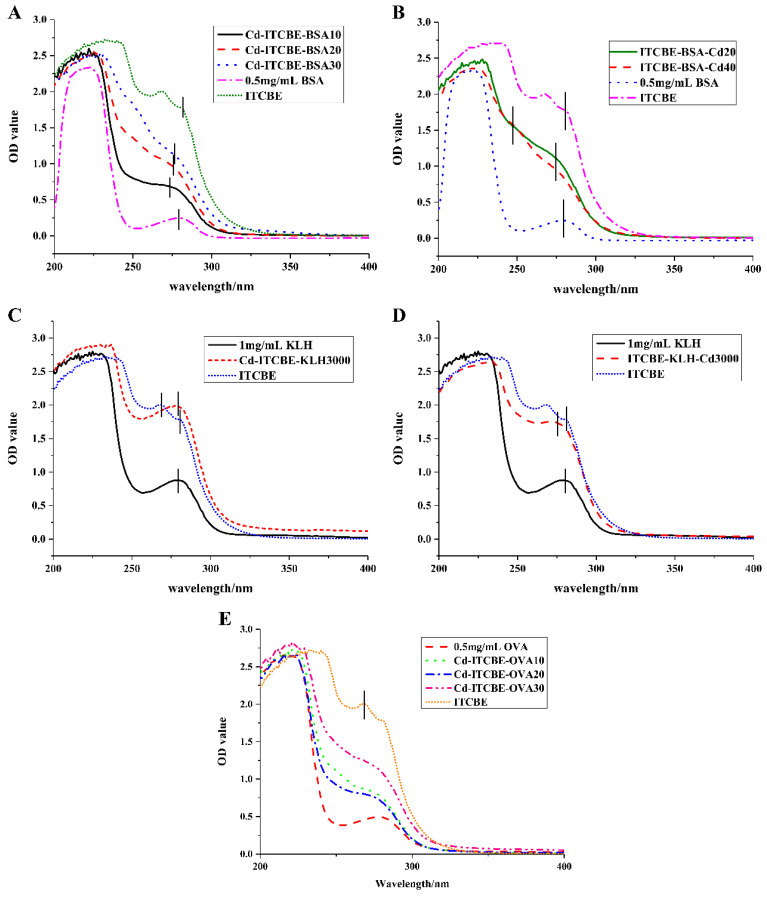
Complete antigens for Cd^2+^ were identified by ultraviolet spectrophotometry. (**A**) The immune antigens against Cd^2+^ based on BSA and method 1; (**B**) The immune antigens against Cd^2+^ based on BSA and method 2; (**C**) The immune antigens against Cd^2+^ based on KLH and method 1; (**D**) The immune antigens against Cd^2+^ based on KLH and method 2; (**E**) Coating antigens for Cd^2+^.

ITCBE belongs to a micromolecular substance (439.44 Da) and does not have immunogenicity. The chelation by Cd^2+^ and ITCBE (Figure S2) can form a stable hexadentate coordination compound (25 °C, 6 h), which has a unique spatial configuration (equivalent to a hapten), and then the chelate is coupled to the carrier protein to prepare an artificial antigen. The common carrier proteins including BSA and KLH were coupled with ITCBE (bifunctional chelator) to synthesize immune antigens, and OVA was coupled to ITCBE to synthesize detection antigens. Ten complete antigens with different concentrations were obtained by methods 1 and 2 (Figure 2). Figure 2 provides the UV spectra of the complete antigen, carrier protein, and chelator at 200–400 nm. As can be seen, both BSA and KLH had typical protein absorption peaks near 280 nm, and ITCBE has two characteristic absorption peaks between 255 and 290 nm. Among the conjugated immunogens and coatings, except for Cd-ITCBE-KLH3000 (Figure 2C), the remaining nine immunogens or coating antigens all had a significant blue shift in UV absorption spectra compared to KLH, BSA and ITCBE (Figure 2A,B,D,E), which indicated the successful synthesis of these nine immunogens or coating antigens [34].

**Figure 3:**
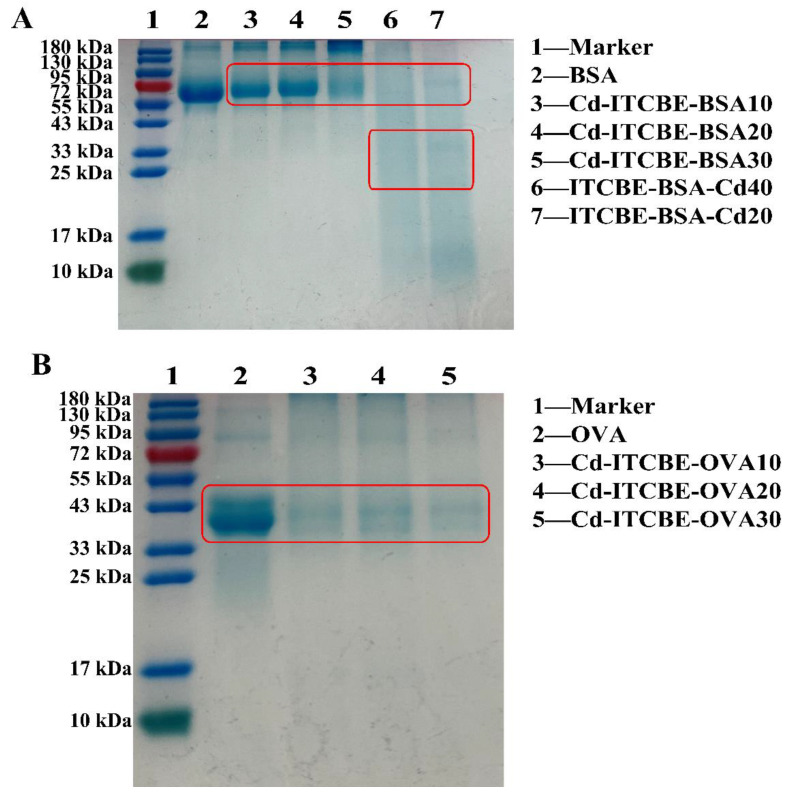
Complete antigens for Cd^2+^ were identified by SDS-PAGE. (**A**) The immune antigens against Cd^2+^ based on BSA; (**B**) Coating antigens for Cd^2+^ based on OVA.

Owing to the combination of the hapten ITCBE, the molecular weight of the synthesized antigen will be larger than that of the carrier protein alone and the migration position will be different. Here, it needs to be explained that the complete antigen synthesized by coupling KLH cannot be identified by SDS-PAGE because the molecular weight of KLH protein is too large. SDS-PAGE analysis (Figure 3) reveals that the electrophoretic bands of Cd-ITCBE-BSA10, Cd-ITCBE-BSA20, and Cd-ITCBE-BSA30 were shifted up compared to the electrophoretic bands of BSA, with the molecular weight close to 66 kDa, indicating that these three immunogens were successfully synthesized. In addition, ITCBE-BSA-Cd20 and ITCBE-BSA-Cd40 showed two electrophoretic bands (25–35 kDa and 66 kDa) and ITCBE-BSA-Cd20 (lower concentration of ITCBE) showed a clearer band at 66 kDa than ITCBE-BSA-Cd40, which may be due to the destruction of the synthetic immunogenic protein in the presence of ITCBE. Likewise, the electrophoretic bands of the three coating antigens, including Cd-ITCBE-OVA10, Cd-ITCBE-OVA20, and Cd-ITCBE-OVA30, were shifted up compared with that of OVA. Combining UV spectroscopy and SDS-PAGE analysis, our results show that the remaining nine complete antigens for Cd^2+^ were successfully synthesized except for Cd-ITCBE-KLH3000.

### 3.3. Animal Immunity and Screening of Positive Cells

**Figure 4:**
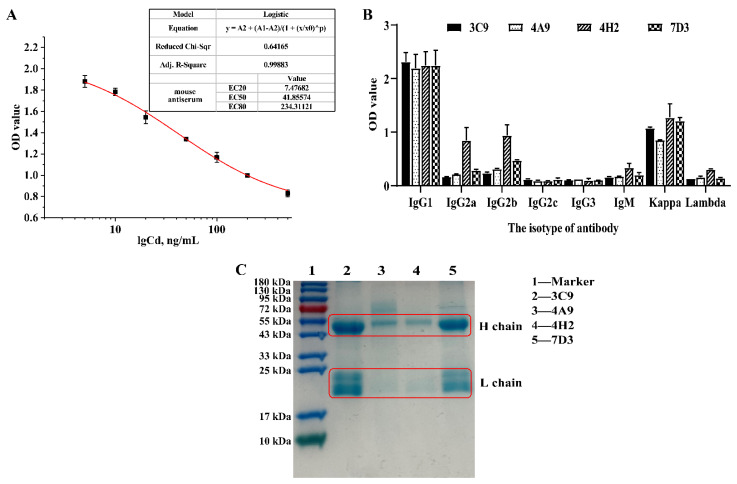
Identification of positive hybridoma cells. (**A**) Standard curve of antiserum obtained after final immunization against immunogen Cd-ITCBE-BSA30 in BALB/c mice. (**B**) Identification of antibody isotype secreted by different cell lines; (**C**) Analysis of the purity of antibodies secreted by different cell lines.

In the current work, the remaining nine synthetic immunogens except Cd-ITCBE-KLH3000 were injected into BALB/c mice. After six immunizations, the titer of the specific antibody in the serum of the immunized mice was detected by the ic-ELISA. It was found that the immunogen Cd-ITCBE-BSA30 showed the highest titer (much higher than 1.6 × 10^5^), indicating that the serum might contain specific mAbs against Cd^2+^ [28]. Besides, Cd-ITCBE-OVA20 was used as the coating antigen and the IC50 of the mouse antiserum measured after the final immunization by ic-ELISA was 41.86 ng/mL, the LOD (IC10, similarly hereinafter) was 5.87 ng/mL, and the measurement range was 7.47–234.31 ng/mL (IC20-80, similarly hereinafter). Compared with the antiserum obtained after the fifth immunization, IC50 was reduced by two times (Figure 4A). The positive hybridoma cells were positively screened by ic-ELISA. After four subclonal screening, four positive cells that could stably secrete mAbs were obtained, namely 3C9, 7D3, 4H2, and 4A9, respectively.

### 3.4. Characterization of Specific mAbs against Cd^2+^

After purification of the ascites, the results of antibody identification show that the above four antibody subtypes secreted by 3C9, 4A9, 4H2, and 7D3 had heavy chains of IgG1 and light chains of Kappa (Figure 4B). It is reported that IgG antibodies can be stably secreted by cells and show high titer for a long time [40]. SDS-PAGE analysis (Figure 4C) showed that the bands of the light chain and heavy chain for the obtained four mAbs were clear, the molecular weight of the heavy chain was nearly 50 kDa, and the light chain was nearly 20 kDa, indicating that the four antibodies had high purity. The concentrations of mAbs secreted by 3C9, 4A9, 4H2, and 7D3 were 3.6, 2.0, 1.8, and 1.6 mg/mL, respectively, which showed that four mAbs had a high yield. Thereinto, the titer of the antibody secreted by 3C9 and 4A9 was higher than 7.29 × 10^5^, whereas the titer of 4H2 and 7D3 was 2.43 × 10^5^. Our results indicate that the four cell lines could produce sufficiently high-purity target antibodies.

**Figure 5:**
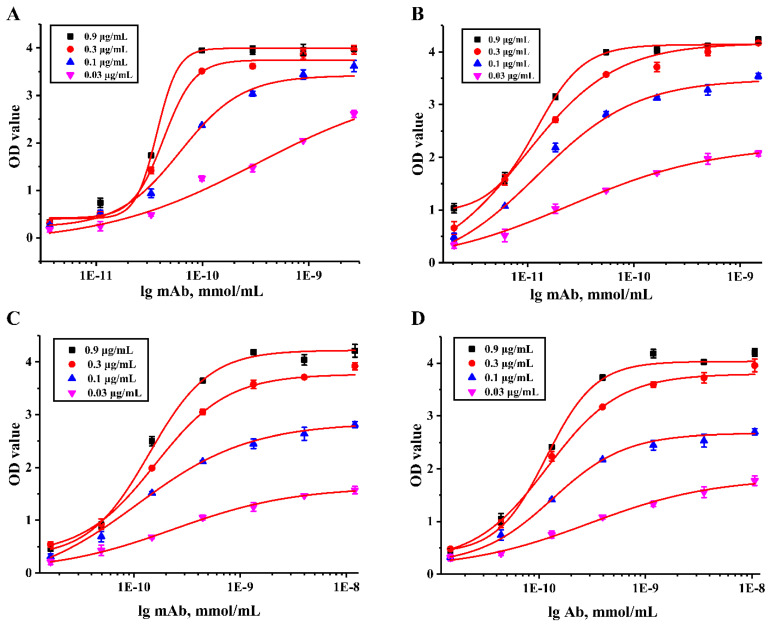
Affinity constants of mAbs secreted by 4 cell lines. (**A**) 3C9; (**B**) 4A9; (**C**) 4H2; (**D**) 7D3. Different colors represent different concentrations of coated antigen.

**Figure 6:**
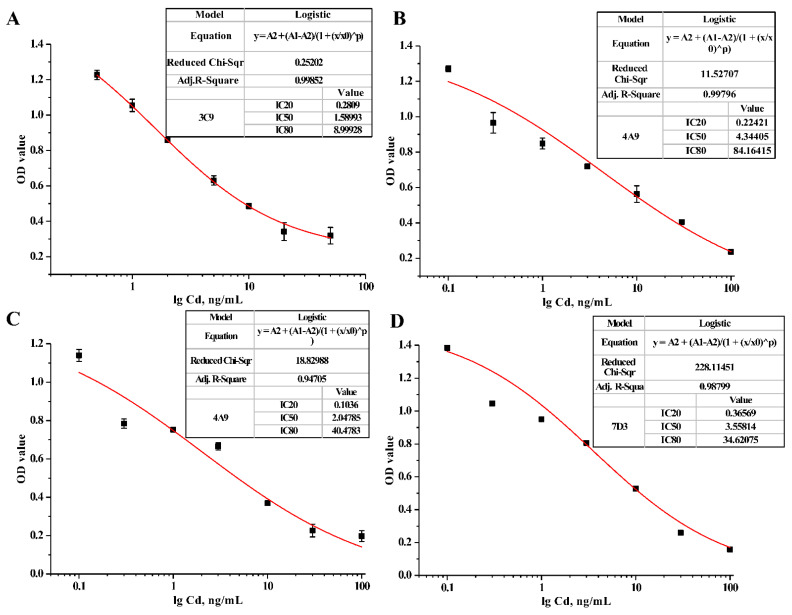
Standard curves of different mAbs for Cd^2+^ detection by ic-ELISA. (**A**) 3C9; (**B**) 4A9; (**C**) 4H2; (**D**) 7D3.

In addition, the affinity constant (Ka) reflecting the binding strength of the antibody to the monovalent epitope was determined by the ic-ELISA method [41]. Different concentrations of Cd-ITCBE-OVA20 coated antigen (0.03, 0.1, 0.3, and 0.9 μg/mL) and a series of diluted and purified mAbs were used in this assay. According to the results obtained in Figure 5, the Ka values of the mAbs secreted by 3C9, 4A9, 4H2, and 7D3 were calculated to be 1.83 × 10^10^, 3.03 × 10^10^, 3.27 × 10^9,^ and 3.30 × 10^9^ L/mol, respectively, which showed that the 4 mAbs prepared had a strong affinity (the Ka above 10^9^). Moreover, the Ka values of the four antibodies were all greater than those of the mAbs against Cd^2+^ previously reported by literature [28], further confirming that four mAbs prepared were high-affinity antibodies. Figure 6 provides the standard curves of 4 mAbs against Cd^2+^. As can be seen from Table 1, The IC50 of the mAb secreted by 3C9 was 1.59 ng/mL, the LOD was 0.13 ng/mL, and the linear detection range (LDR) was 0.28–9.00 ng/mL; the IC50 of the mAb secreted by 4A9 was 4.34 ng/mL, the LOD was 0.04 ng/mL, and the LDR was 0.22–84.16 ng/mL; the IC50 of the mAb secreted by 4H2 was 2.05 ng/mL, the LOD was 0.02 ng/mL, and the LDR was 0.10–40.48 ng/mL; the IC50 of the mAb secreted by 7D3 was 3.56 ng/mL, the LOD was 0.10 ng/mL, and the LDR was 0.37–34.62 ng/mL. To analyze the specificity of the obtained mAbs, the cross-reaction rates of 4 mAbs were evaluated for different heavy metal ion chelates that bind to EDTA, including EDTA-Hg, EDTA-Pb, EDTA-As, EDTA-Cu, EDTA-Fe, EDTA-Ni, and EDTA-Cr. The results of ic-ELISA analysis (Table 2) show that the four mAbs had a certain cross-reactivity with EDTA-Hg, and the cross-reactions with the other six EDTA-metal ions were all less than 1%, this result was consistent with the previous report of Blake et al. [42]. Studies have shown that the chelator and metal ions were combined through chemical bonds to change the configuration of the chelator, and the chelator binding to different metal ions has different configurations [43]. The configuration of the chelator is an important factor for determining the recognition of antigen-antibody. Jones et al. used the distance matrix method to compare the three-dimensional structure differences of different metal chelate molecules and found that the three-dimensional structure differences between Cd-EDTA and Hg-EDTA were the lowest, which was the main reason for the cross-reaction [44]. Our results indicate that the prepared four mAbs had strong specificity.

Since the mAb secreted by 3C9 had a stronger affinity, the lowest IC50, and the stronger specificity, it was chosen as the antibody against Cd^2+^ for the development of immunochromatographic test strips.

### 3.5. Preparation of Latex Microsphere Immunochromatographic Test Strips for Detection of Cd^2+^

In addition to its theoretical research, mAbs are widely used in in vitro diagnosis and disease treatment [45]. In food safety research, mAbs are often used in the detection of toxic and harmful substances, whereas immunochromatographic assay has become the main detection method in rapid detection field because of its advantages such as rapid detection, high efficiency and easy operation. The most common is the color-indicated immunochromatographic detection technology [46]. Bioassays using immunochromatography can easily achieve on-site measurement of samples without using expensive equipment. Traditional color-indicated immunochromatographic assay has some shortcomings that cannot be ignored, which limits its large-scale on-site application. For example, the sensitivity of colloidal gold immunochromatographic test strips is often affected by its signal molecule (colloidal gold) [47]. Additionally, immunochromatographic test strips based on SERS and fluorescence usually need instrument assistance for more sensitive readings and cannot be used for large-scale applications [48]. Latex microspheres, as the tracer of immunochromatographic test strips, have uniform particle size (Figure S3) and strong stability and can provide more sensitive detection results without the instrument assistant [28]. At present, the popular immunochromatographic test strips in the market mostly use colloidal gold particles. Compared with colloidal gold, the red latex microspheres we use have larger particle size, larger surface area, and stronger capability to bind to antibodies, as well as higher sensitivity [24,46]. Studies have reported that the LMIA method was applied to detect anti-*Schisaosoma japonicum* antibodies in human serum. In addition, the LMIA method has also been developed to quantitatively detect dexamethasone in milk and pork [49]. However, the research on LMIA method for Cd^2+^ detection has not been reported. Thus, based on the current traditional instrument-assisted detection method of heavy metal ions in asparagus, the novel LMIA technology for rapid detection of Cd^2+^ in asparagus was developed.

**Figure 7:**
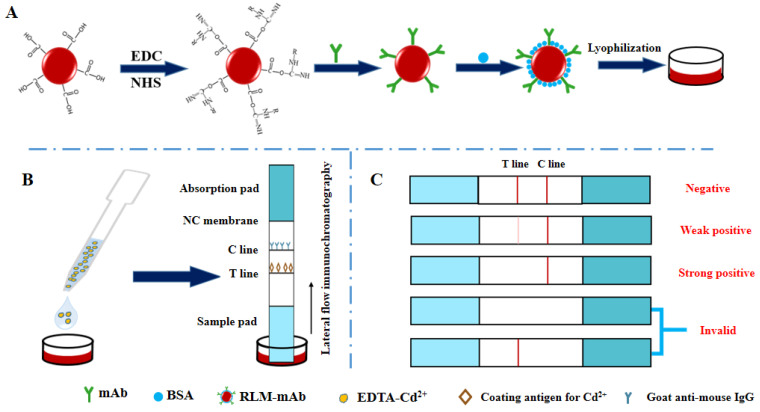
Schematic diagram of the preparation of immunochromatographic microsphere test strips for the Cd^2+^ detection. (**A**) Probe preparation; (**B**) Operation of test strip; (**C**) Judgment of the result displayed by the test strip.

The activated latex microspheres were mixed with 200 μg of mAb secreted by 3C9, and then a 5% BSA solution was added to construct a detection probe (Figure 7A). The maximum absorption peak of the prepared probe had both the absorbent characteristics of latex microspheres and mAb and had obvious shifting (Figure S4), indicating that the probe was successfully synthesized and could be used for subsequent Cd^2+^ detection. The coating agent (0.09 mg/mL Cd-ITCBE-OVA20) was sprayed on the T line and the goat anti-mouse IgG antibody (1 mg/mL) was sprayed on the C line. The test strip device contained a nitrocellulose (NC) membrane and two pads (absorption and sample) (Figure 7B). If T and C lines are both colored, it means that there is no Cd^2+^ in the sample; if the T line is not colored and the C line is colored, indicating that there is Cd^2+^ in the sample; if the C line is not colored, no matter whether the T line is colored or not, indicating that the test strip is invalid (Figure 7C) [21,49,50]. The results could be evaluated visually within 3–5 min.

### 3.6. Sensitivity Evaluation of Latex Microsphere Immunochromatographic Test Strips

**Figure 8:**
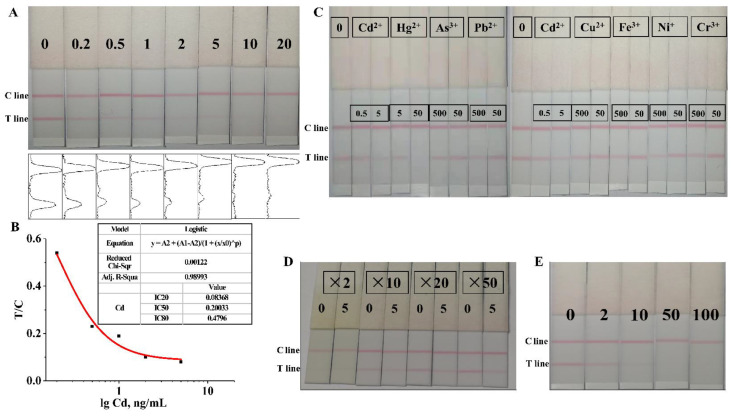
Characterization of immunochromatographic test strips based on the mAb for cadmium ion, concentration unit, ng/mL. (**A**) Sensitivity of test strips; (**B**) Standard curve of test strip sensitivity; (**C**) Specificity of test strips; (**D**) Influence of sample matrix effect on test strip (‘×’ represent dilution multiple); (**E**) Measurement of actual samples.

To evaluate the sensitivity of the obtained test strips, different concentrations of EDTA-Cd (0, 0.2, 0.5, 1, 2, 5, 10, and 20 ng/mL) were assayed to reflect the LOD of the test strips. As shown in Figure 8A, when 0.2 ng/mL of EDTA-Cd was added, the color on the T line of the test strip changed significantly, while the color on the T line completely disappeared with the addition of 5 ng/mL EDTA-Cd, indicating that the visual LOD of test strip was 0.2 ng/mL and the linear elimination value was 5 ng/mL. The ImageJ (Version 1.8.0) software was employed to record the gray values of the T and C lines of the obtained test strip. A standard curve was established with the log value of the Cd^2+^ concentration as the abscissa and the gray value of the T/C line as the ordinate (Figure 8B). The lowest LOD could reach 0.054 ng/mL (with IC10 as the LOD), IC50 was 0.2 ng/mL, and the LDR was 0.08–0.48 ng/mL. In addition, the analytical property of the prepared LMIA test strip had been compared with other reported measurement methods for Cd^2+^ detection (Table 3). Comparative analysis reveals that the established LMIA test strip exhibited better performance in terms of test time and sensitivity, which further shows that the developed LMIA test strips seem to be sufficient as a rapid and effective tool for the rapid monitoring and high throughput screening of Cd^2+^ in asparagus.

### 3.7. Specificity Evaluation of Latex Microsphere Immunochromatographic Test Strips

The specificity of immunochromatographic test strips is often related to the specificity of the antibody used. The result obtained from Figure 8C shows that the test strip had a certain cross-reaction with Hg^2+^ and had no cross-reaction with other heavy metal ions, which was in line with the test results of mAb specificity and exhibited the strong specificity of the test strip.

### 3.8. Detection of Cd^2+^ in Asparagus Samples

To determine the matrix effect of the asparagus sample on the test strip, the pre-treated asparagus samples were diluted 2, 10, 20, and 50 times with HBS-EDTA solution. The sample solution diluted two times was yellow (Figure S5), and the measurement result of the corresponding test strip (Figure 8D) shows that there was no difference in the color of the T line between 0 ng/mL Cd^2+^ and 5 ng/mL Cd^2+^, indicating that the matrix effect of the sample solution diluted two times had a great impact on the test results. Similarly, the matrix effect of the sample solution diluted 10 times also had a certain influence on the test results. However, the matrix effect of the sample solution diluted 20 times and 50 times had no significant effect on the test results. Our results show that when the sample solution was diluted 20 times, the influence of the asparagus matrix effect on the test strip test results could be almost eliminated.

To determine the LOD of the prepared test strips for the detection of Cd^2+^ in asparagus, a series of EDTA-Cd solutions (2, 10, 50, and 100 ng/mL) were prepared for the test strip assay. It can be seen from Figure 8E that the addition of 2 ng/mL EDTA-Cd caused the color on the T line significantly different compared with the addition of 0 ng/mL EDTA-Cd, whereas the color on the T line disappeared completely with the addition of 50 ng/mL EDTA-Cd. This result revealed that the visual LOD of the test strip in the Cd^2+^ detection in asparagus was 2 ng/mL and the linear elimination value was 50 ng/mL, which met the limit standard of the national standard of China (GB 2762-2017). The Cd^2+^ content of the asparagus sample used was verified as a negative sample by using AAS recommended by the national standard of China, which was consistent with the test result of the test strip, indicating that the result was accurate.

## 4. Conclusions

We report a novel LMIA method to rapidly detect Cd^2+^ in asparagus. Through the growth of the *pca1*Δ yeast strain, the chelating capability of various chelators to Cd^2+^ was systematically analyzed. In addition, based on the obtained mAb with high affinity and high specificity, the LOD of the developed LMIA test strip was 0.054 ng/mL, the visual LOD was 0.2 ng/mL, and the LDR was 0.08–0.48 ng/mL. In the detection of actual asparagus samples, the visual LOD was 2 ng/mL and the linear elimination value was 50 ng/mL. Therefore, this rapid and effective tool can be widely used in the analysis of Cd^2+^ residues in a large number of on-site asparagus samples, and even provides potential applications for the detection of Cd^2+^ in vegetable products.

## Figures and Tables

**Table 1:** Comparison of detection characteristics of monoclonal antibodies secreted by four cell lines.

Cell Strain	IC50 (ng/mL)	LOD (ng/mL)	Linear Range (ng/mL)
3C9	1.59	0.13	0.28–9.00
4A9	4.34	0.04	0.22–84.16
4H2	2.05	0.02	0.10–40.48
7D3	3.56	0.10	0.37–34.62

**Table 2:** Cross-reaction rate of four mAbs for EDTA-metal ions.

mAb Number	3C9		4A9		4H2		7D3	
EDTA-Meta Ions	IC_50_, ng/mL	CR%	IC_50_, ng/mL	CR%	IC_50_, ng/mL	CR%	IC_50_, ng/mL	CR%
EDTA-Cd	1.61 ± 0.01	100	4.32 ± 0.03	100	2.01 ± 0.02	100	3.62 ± 0.03	100
EDTA-Hg	>10	<16%	>50	<8%	>50	<4%	>10	<36%
EDTA-Pb	>500	<0.3%	>500	<0.8%	>500	<0.4%	>500	<0.7%
EDTA-As	>500	<0.3%	>500	<0.8%	>500	<0.4%	>500	<0.7%
EDTA-Cu	>500	<0.3%	>500	<0.8%	>500	<0.4%	>500	<0.7%
EDTA-Fe	>500	<0.3%	>500	<0.8%	>500	<0.4%	>500	<0.7%
EDTA-Ni	>500	<0.3%	>500	<0.8%	>500	<0.4%	>500	<0.7%
EDTA-Cr	>500	<0.3%	>500	<0.8%	>500	<0.4%	>500	<0.7%

**Table 3:** Comparison with the reported Cd^2+^ immunoassays.

Methods	Linear Detection Range (ng/mL)	IC50 (ng/mL)	Detection Limit (ng/mL)		Detection Time	Reference
			vLOD	LOQ		
ic-ELISA	-	45.6	-	1.953	>2 h	[51]
ic-ELISA	0.1–1000	-	-	0.1	>2 h	[52]
ic-ELISA	0.2–40	2.59	-	0.08	>3 h	[53]
Silver-enhanced ICA strip	0.5–5	-	5	0.35	>30 min	[23]
Competitive ICA strip	0.25–8	-	-	0.18	1 h	[24]
Fuorescent immunoassay strip	3.8–48.9	-	-	1.93	12 min	[54]
LMIA strip	0.08–0.48	0.20	2.00	0.05	3–5 min	this work

## Data Availability

The datasets used and/or analyzed during the current study are available from the corresponding author on request.

## Supplementary Materials

The following are available online at https://www.mdpi.com/article/10.3390/foods11010078/s1, Figure S1: Structural formulas of 8 chelating agents, including 5-Br-PADAP, 2-MBT, NAC, CADION, dimercaptosuccinic acid, CaNa2-EDTA, EDTA, and ITCBE, Figure S2: Synthetic principle of complete immune antigen for Cd^2+^, Figure S3: SEM image of red latex microspheres, Figure S4: UV spectra of mAb, latex microspheres, and mAb-latex microspheres, Figure S5: Color of pretreated asparagus samples after different dilutions (×2, ×5, ×10, and ×50), Table S1: Reagents and chemicals.
