# Supplementary material for: Novel Latex Microsphere Immunochromatographic Assay for Rapid Detection of Cadmium Ion in Asparagus
Source: Foods. 2021 Dec 29;11(1):78. doi: 10.3390/foods11010078 (PMC8750861; doi:10.3390/foods11010078)
Supplement: Supplementary file 1 [file foods-11-00078-s001.zip › foods-1467471-SI.pdf]

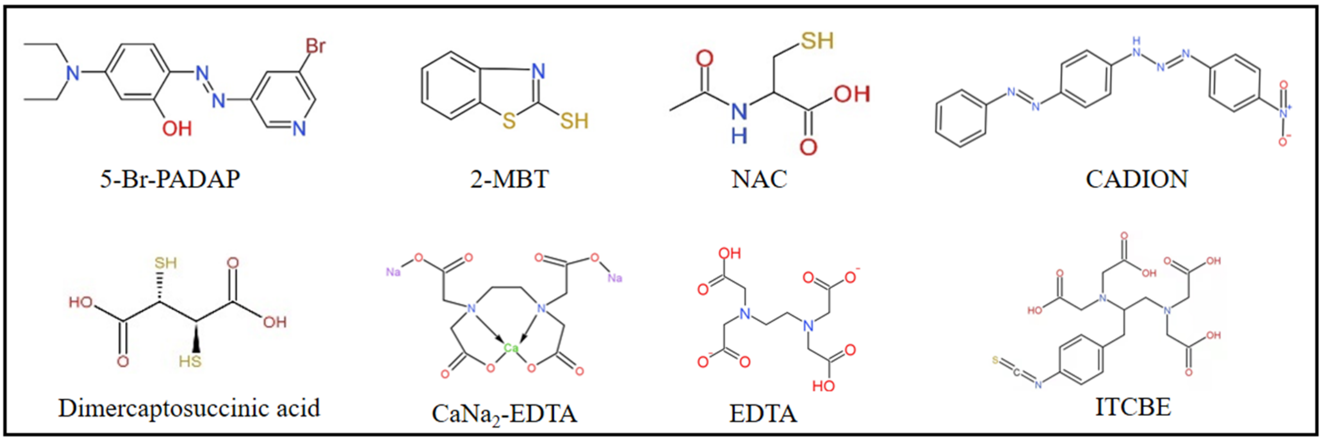

Figure S1. Structural formulas of 8 chelating agents, including 5-Br-PADAP, 2-MBT, NAC, CADION, dimercaptosuccinic acid, $CaNa_2$-EDTA, EDTA, and ITCBE.

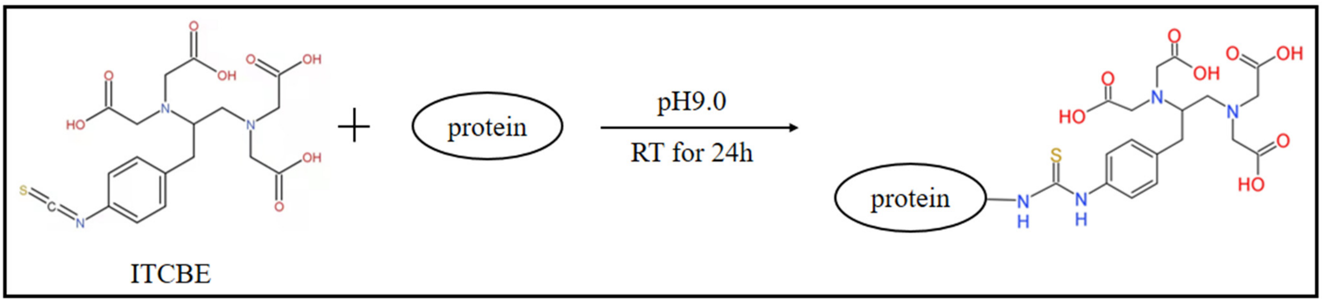

Figure S2. Synthetic principle of complete immune antigen for Cd^{2+}.

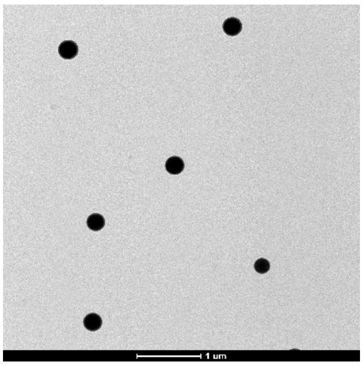

Figure S3. SEM image of red latex microspheres.

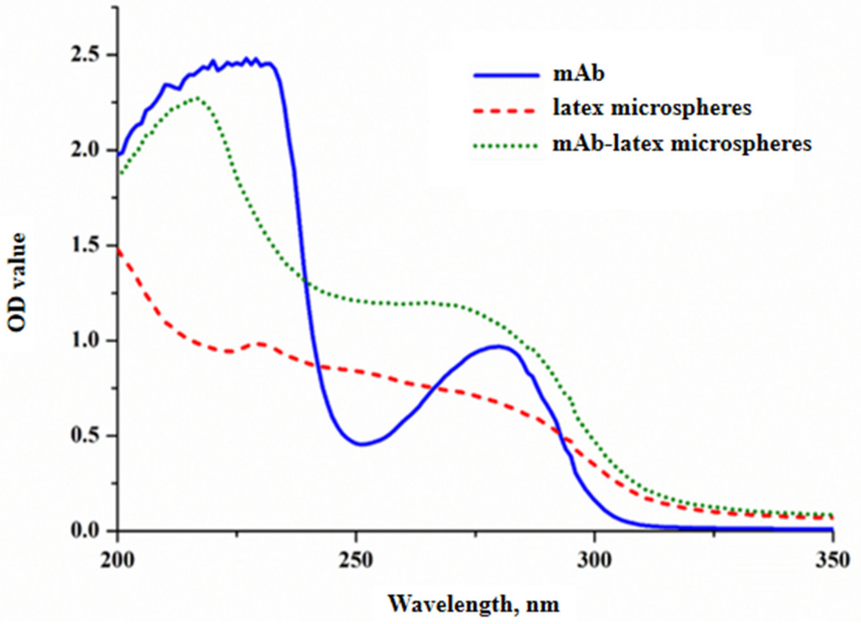

Figure S4. UV spectra of mAb, latex microspheres, and mAb-latex microspheres.

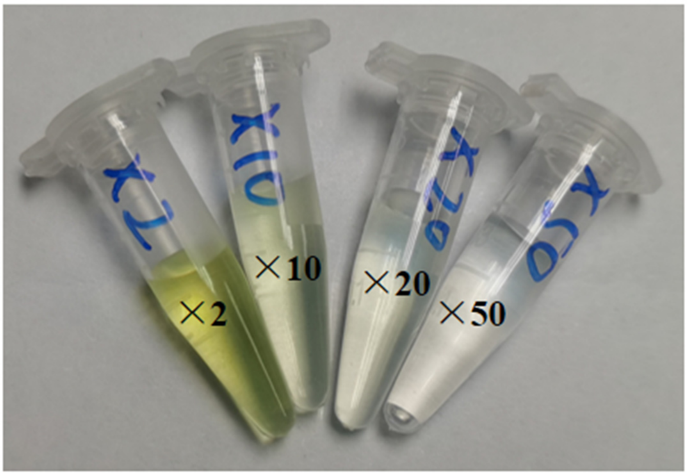

Figure S5. Color of pretreated asparagus samples after different dilutions (×2, ×5, ×10, and ×50).

Table S1. Reagents and chemicals

Chemicals	Manufacturers	Locations
$CdCl_2$	Sinopharm Chemical Reagent Co., Ltd.	Shanghai, China
ITCBE	Dojindo	Kyushu, Japan
$Cd(NO_3)_2$	National Standard Substances Center of China	Beijing, China
5-Br-PADAP, 2-MBT, NAC, CADION, dimercaptosuccinic acid, $CaNa_2$-EDTA, and EDTA	Macklin	Shanghai, China

Ovalbumin (OVA), bovine serum albumin (BSA), and keyhole limpet hemocyanin (KLH)	Sigma Co.	St Louis, USA
Mouse mAb subtype identification (MAI) ELISA kit	Proteintech	Pennsylvania, USA
Red carboxyl latex microspheres	Suzhou Vdo Biotech Co., Ltd.	Suzhou, China
HRP-conjugated antibody and Goat anti-mouse IgG antibody	Cellwaylab	Luoyang, China
